# Efficient Energy Transfer in Eu^3+^-Doped Layered Double Hydroxides with β‑Diketonate Anions Obtained by the Memory Effect

**DOI:** 10.1021/acsomega.5c05499

**Published:** 2025-09-17

**Authors:** Alexandre Candido Teixeira, Natan Felipe Netzlaff Fachini, Henrique Kenzo Carvalho Kakinami, Danilo Mustafa

**Affiliations:** 61755 Instituto de Física da Universidade de São Paulo, 05508-090 São Paulo, SP, Brazil

## Abstract

The use of layered double hydroxides (LDHs) as luminescent hybrid materials has gained attention due to their structural versatility and ability to incorporate functional anions. In this study, we report the synthesis of a ZnAlEu–LDH intercalated with dibenzoylmethane (DBM) via the memory effect as an alternative strategy to obtain luminescent materials. The LDH precursor was synthesized by coprecipitation and then calcined at 460 °C to form a mixed oxide, followed by structural reconstruction in an aqueous DBM solution. The structural transformation and intercalation processes were confirmed by powder X-ray diffraction, Fourier-transform infrared spectroscopy, and elemental analysis (CHN and inductively coupled plasma optical emission spectrometry). Morphological changes were evaluated by scanning electron microscopy. Photoluminescence spectroscopy revealed that the DBM-intercalated LDHs exhibited a significant enhancement in the Eu^3+^ emission intensity due to the antenna effect from DBM, as demonstrated by the appearance of a broad S_0_(π) → S_*n*_(π*) excitation band around 390 nm. The emission spectra also showed characteristic Eu^3+^ transitions with spectral shifts indicative of changes in the local ligand field upon DBM coordination. This work demonstrates that the memory effect is an effective strategy for incorporating photofunctional ligands into LDH matrices, opening new possibilities for the design of luminescent hybrid materials with tailored properties.

## Graphical abstract

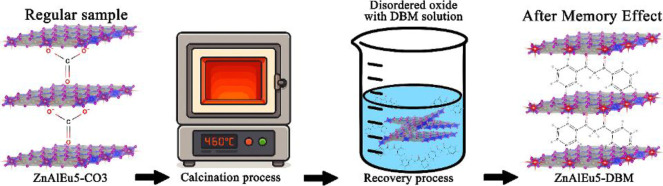

## 1 Introduction

Layered double hydroxides (LDHs) are an important class of bimetallic hydroxide materials with general formula ${{[M}_{1-x}^{2+}M_{x}^{3+}{\left(OH\right)}_{2}]}^{x+}A_{x/m}^{m-}nH_{2}O$ , where M^2+^ and M^3+^ represent divalent and trivalent metal cations, respectively, and A^*n*–^ denotes interlayer anions (Figure 1) The isomorphic substitution of M^2+^ by M^3+^ introduces positive charges in the hydroxide layers, which are balanced by the intercalation of anions in the interlayer spaces. This structural versatility allows for the fine-tuning of LDH properties, making them suitable for applications in catalysis, drug delivery, environmental remediation, and as luminescent materials.^1–4^

**Figure 1:**
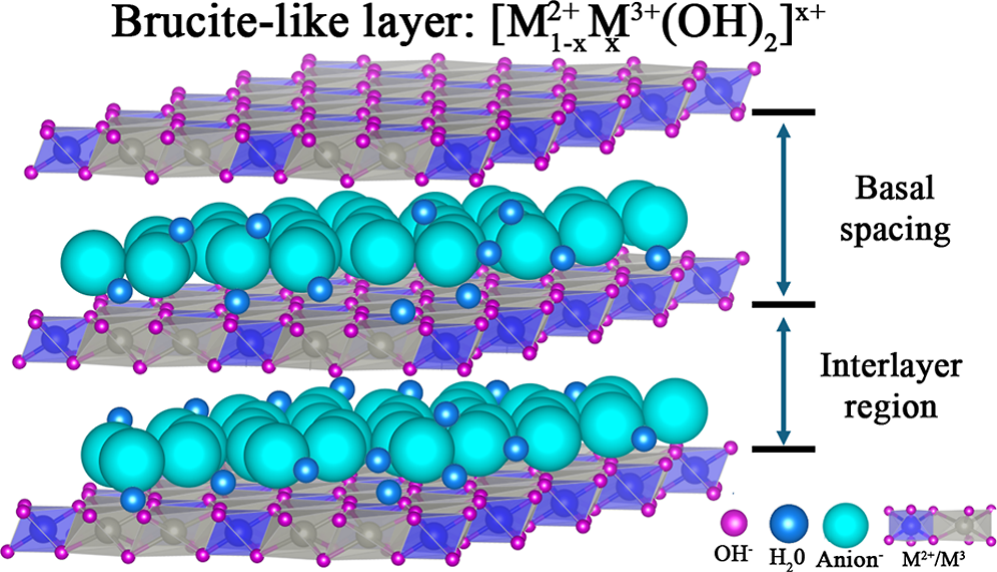
Schematic representation of the LDH structure. The figure illustrates the brucite-like metal hydroxide layers, the interlayer region containing charge-balancing anions, and water molecules. Metal cations M^2+^ and M^3+^ are octahedrally coordinated with hydroxide ions.

Incorporating trivalent rare-earth ions (RE^3+^), such as europium (Eu^3+^), into LDH matrices has led to the development of two-dimensional materials with remarkable luminescent properties.^5–7^ Rare-earth elements, which belong to group 3 of the periodic table, are characterized by optical transitions that are largely unaffected by the surrounding ligand field. However, these transitions are Laporte-forbidden, resulting in low molar absorption coefficients.^8–11^ To overcome this limitation and enhance the emission efficiency, it is common to embed RE^3+^ ions in host matrices capable of efficiently absorbing light and subsequently transferring energy to the lanthanide centers. This energy transfer mechanism, commonly referred to as the “antenna effect”, significantly increases the population of excited states in the RE^3+^ ions, thereby amplifying their luminescence output.^8,12,13^

Previous studies have demonstrated the successful incorporation of anionic sensitizers, such as carboxylates and dicarboxylates, into LDHs via direct coprecipitation or postsynthetic anion exchange. For instance, the intercalation of 4-biphenylacetate and sulfonates has facilitated efficient energy transfer to RE^3+^ ions within the hydroxide layers. Notably, some research has shown that both the ligand and the RE^3+^ ion can reside within the interlayer space, enhancing luminescent properties through close proximity.^10,14,15^

The “memory effect” of LDHs refers to the ability to reconstruct their original lamellar structure upon rehydration after thermal decomposition (Figure 2). Controlled thermal decomposition of LDHs leads first to the formation of an amorphous mixed metal oxyhydroxide phase before transitioning into the fully crystalline oxide.^16^ During calcination, typically between 350 and 500 °C, interlayer anions (such as carbonate) are eliminated, along with the removal of interlayer water and partial dehydroxylation of the hydroxide layers. The resulting disordered oxide retains a high surface reactivity, enabling the restoration of the lamellar structure when exposed to aqueous solutions containing appropriate anionic species.^17,18^

**Figure 2:**
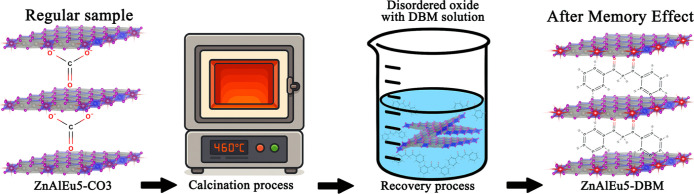
Schematic illustration of the structural transformation of ZnAlEu5-CO_3_ LDH during the calcination and reconstruction processes via the memory effect. The loss of carbonate and water upon calcination and the subsequent intercalation of dibenzoylmethane (DBM) anions during rehydration are represented.

This phenomenon not only enables the structural reconstruction of LDHs but also provides a powerful route for the selective incorporation of functional anions during the rehydration process. While the memory effect has been widely explored for applications in environmental remediation, particularly for the removal of anionic pollutants, its use in incorporating photofunctional organic ligands remains comparatively underexplored.^19,20^

While previous works have successfully incorporated simple carboxylate ligands into LDHs via coprecipitation or ion-exchange routes, these methods often face limitations related to ligand solubility, steric hindrance, and competitive phase formation. In contrast, the memory effect offers a robust and versatile pathway for introducing bulky or hydrophobic functional anions, such as photofunctional β-diketonates, directly into the interlayer space while preserving the layered structure. Teixeira et al.^17^ have shown that the memory effect can be used to intercalate the 1,3,5-benzenetricarboxylate (BTC, tricarboxylate anion) photosensitizers between the interlayer space of the HDLs, providing a useful tool to monitor their rehydration process. Despite its widespread application in environmental remediation, the potential of the memory effect for the fabrication of luminescent hybrid LDHs remains largely unexplored.

DBM is a β-diketone ligand, which combines strong chelating affinity for lanthanide ions with intense S_0_(π) → S_*n*_(π*) transitions, making it highly effective in sensitizing Eu^3+^ luminescence through the antenna effect.^21^ Its molecular structure promotes an efficient absorption of ultraviolet light, followed by nonradiative energy transfer to the Eu^3+^ excited states, ultimately enhancing the characteristic red emission of Eu^3+^.

In this work, we explore an alternative and underutilized strategy for synthesizing luminescent LDHs by employing the memory effect to replace carbonate anions with DBM. The approach involves the thermal decomposition of [Zn_2_Al_0.95_Eu_0.05_(OH)_6_]·(CO_3_^2–^) at 460 °C, followed by a controlled rehydration process in DBM-containing aqueous solutions. This enables the reconstruction of the LDH structure while incorporating DBM ligands within the interlayer space, an intercalation pathway made possible only via the memory effect due to DBM’s low solubility and steric bulk, which prevent its insertion through conventional coprecipitation or ion-exchange routes. Once intercalated, DBM molecules act as antenna sensitizers, efficiently transferring energy to Eu^3+^ ions embedded in the hydroxide layers and leading to enhanced luminescence.

Beyond the introduction of a novel organic ligand, the present study expands the characterization methodology by including scanning electron microscopy (SEM), Fourier-transform infrared (FTIR), inductively coupled plasma optical emission spectrometry (ICP-OES), CHN, and Judd–Ofelt analysis (Ω_2_), allowing for a detailed correlation between the Eu^3+^ coordination environment, structural distortion, and photophysical behavior. Notably, we also examine a partially rehydrated intermediate (60 min) to provide mechanistic insight into the structural evolution and progressive DBM coordination—an aspect not addressed in previous BTC-based systems.^17^ The resulting luminescent hybrid LDH materials exhibit photoluminescence quantum yields that surpass the values typically reported for analogous LDH with Eu^3+^ systems,^21^ further underscoring the effectiveness of the memory effect as a postsynthetic functionalization strategy.

## 2 Materials and Methods

### 2.1 Materials

Zinc nitrate hexahydrate (Zn­(NO_3_)_2_·6H_2_O, 96%, LabSynth), aluminum nitrate nonahydrate (Al­(NO_3_)_3_·9H_2_O, 98%, LabSynth), sodium carbonate (Na_2_CO_3_ 96% LabSynth), dibenzoylmethane (DBM, Vetec), and sodium hydroxide (NaOH, 97 mol %, Vetec) were used without further purification. Eu­(NO_3_)_3_·6H_2_O was synthesized by reacting Eu_2_O_3_ with a nitric acid (CSTARM, 99% China) precursor.^22^

### 2.2 Synthesis

The LDH was synthesized using the coprecipitation method at a constant pH, room temperature, and ambient pressure.^1^ A 10 mL volume of a 1 mol L^–1^ solution containing the metal precursors Zn­(NO_3_)_2_·6H_2_O, Al­(NO_3_)_3_·9H_2_O, and Eu­(NO_3_)_3_·6H_2_O in a 2:0.95:0.05 molar ratio was added dropwise (∼10 mL h^–1^) into 200 mL of deionized water containing 20 mmol of dissolved Na_2_CO_3_. During the synthesis, the solution was continuously stirred, while the pH was maintained at 10 using an automatic titrator (Titrino 702 SM, Metrohm, Switzerland). The resulting suspension was equilibrated in a closed vessel at 60 °C for 24 h, followed by centrifugation and rinsing with distilled water. Finally, the solid phase was air-dried at 60 °C for 3 days.

### 2.3 Calcination

The ZnAlEu-CO_3_ LDH was calcined in a muffle furnace at 460 °C for 1 h (ZAE-460), with the temperature increasing from ambient to the final value at a rate of 5 °C min^–1^.

### 2.4 Rehydration

The rehydrated samples were obtained by suspending 100 mg of the calcined LDHs in 10 mL of a 12 mmol L^–1^ DBM aqueous solution. This solution was prepared by dissolving DBM in heated water (60 °C) and then titrating it with approximately 0.3 mL of a 1 M NaOH solution until it reached pH 12. The rehydration process was carried out under continuous stirring (∼300 rpm), with rehydration times ranging from 1 min to 24 h.

### 2.5 Characterization

Powder X-ray diffraction (PXRD) patterns were recorded in Bragg–Brentano geometry using a D8 Discover diffractometer (Bruker) with Cu Kα radiation (λ = 1.5418 Å, 40 kV, 30 mA) and a Lynxeye detector. Data were collected over a 2θ range of 4–70°, with a step size of 0.02°, an integration time of 1 s per step, and sample rotation at 15 rpm. Elemental analysis (CHN) was performed using a PerkinElmer 2400 Series II elemental analyzer via the Pregl–Dumas method to determine the carbon, hydrogen, and nitrogen content. ICP-OES was carried out using a Spectro Arcos analyzer (SPECTRO Analytical Instruments GmbH, Germany) to quantify the Zn, Al, and Eu content. Thermogravimetric analysis (TGA) was performed on a TGA Q500 instrument (TA Instruments) under an air flow of 60 mL min^–1^, with heating from room temperature to 800 °C at a rate of 5 °C min^–1^. FTIR spectra were collected in the range 400–4000 cm^–1^ using a PerkinElmer Frontier FTIR spectrometer with KBr pellet samples. Photoluminescence excitation and emission spectra were recorded at room temperature using an FS5 spectrofluorometer (Edinburgh Instruments) equipped with a 150 W xenon arc lamp. Measurements were performed in solid state using front-face geometry, with spectral correction applied to both excitation and emission channels.

## 3 Results and Discussion

The XRD patterns of the prepared sample ZAE5-CO_3_ (Figure 3a) exhibit sharp reflections at 2θ = 11.72°, 23.05°, 34.68°, 60.24°, and 61.5°, which are indexed to the (003), (006), (012), (110), and (113) planes, respectively, of the typical hydrotalcite LDH structure with a rhombohedral symmetry (3R polytype, JCDS 0048-1023).^23,24^ No impurity phases were observed.

**Figure 3:**
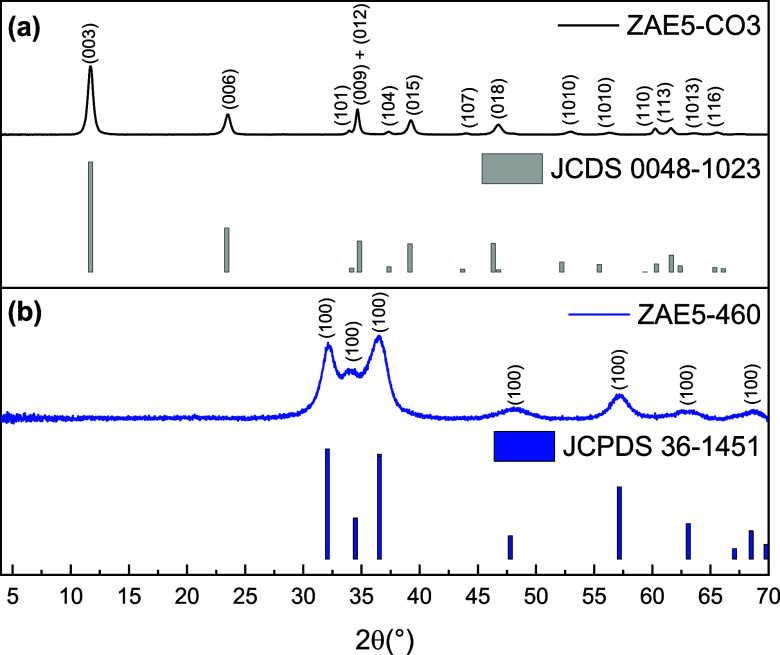
(a) PXRD patterns of ZnAlEu5-CO_3_ (ZAE5-CO_3_) and (b) the corresponding sample after calcination at 460 °C (ZAE5-460). The diffraction peaks of the hydrotalcite-like LDH structure and the transformation into amorphous oxide and ZnO phases are highlighted.

The basal spacing, calculated from the (003) reflection, is 0.756 nm, consistent with the presence of carbonate anions in the interlayer space, as typically observed for carbonate-intercalated LDHs.^5^ Likewise, lattice parameter *a*, which reflects the distance between metal cations in the brucite-like layers, was determined from the (110) reflection to be 0.307 nm. This value indicates the formation of a well-defined ZnAl–LDH structure, with partial incorporation of Eu^3+^ ions, which does not significantly distort the crystal lattice a behavior commonly reported for low Eu doping levels in LDH matrices.^2,3^

Upon calcination at 460 °C (Figure 3b), the characteristic LDH diffraction peaks completely disappear, indicating the collapse of the layered structure.^16,17^ In their place, new broad peaks emerge at 2θ ≈ 31.8°, 34.4°, and 36.3°, which correspond to the (100), (002), and (101) planes of wurtzite-type ZnO (JCPDS 36-1451).^8,25^ The broadening of these peaks suggests the formation of nanocrystalline ZnO domains embedded in an amorphous matrix derived from aluminum oxide species.^26^

This structural transformation is characteristic of the thermal decomposition of carbonate-LDHs, where dehydroxylation and decarbonation processes lead to collapse of the lamellar structure, resulting in mixed metal oxides. In particular, the formation of crystalline ZnO occurs concomitantly with the generation of amorphous alumina, which does not exhibit detectable diffraction peaks in the XRD pattern due to its low long-range order.^17^

No diffraction peaks associated with europium oxides are detected, suggesting that Eu^3+^ ions are either highly dispersed in the amorphous alumina matrix or incorporated into ZnO nanocrystals at concentrations below the XRD detection limit.

To optimize the memory effect and achieve efficient carbonate removal in sample ZAE5-CO_3_, it is essential to select an appropriate calcination temperature based on TGA (Figure 4). The DTG curve reveals four distinct weight loss stages.^27^

**Figure 4:**
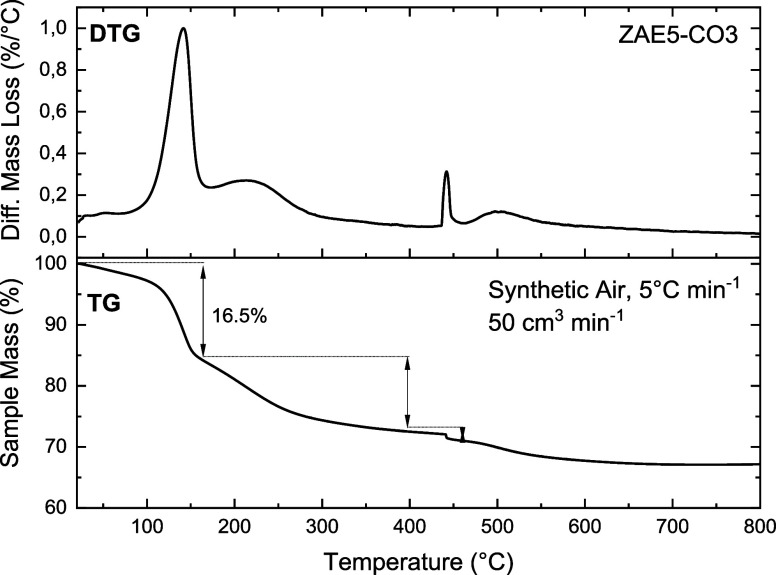
Thermogravimetric analysis (TGA/DTG) of the ZnAlEu5-CO_3_ (ZAE5-CO_3_) sample. The main weight loss events corresponding to water desorption, dehydroxylation, and carbonate decomposition are indicated, supporting the selection of the calcination temperature.

The first, occurring between 80 and 170 °C, is the most prominent and corresponds to the release of the interlayer and surface-adsorbed water. This step is also associated with the collapse of the lamellar structure, a phenomenon observed in Zn/Al LDHs and similarly reported for Eu^3+^-doped LDHs.^17^

The second stage, between 170 and 230 °C, is attributed to the onset of dehydroxylation of the hydroxide layers.^28,29^

The third stage, from 350 to 500 °C, is characterized by a significant mass loss centered around 441 °C, corresponding to the complete dehydroxylation of the layers and the decomposition of interlayer carbonate species.^16^ At this point, the lamellar structure irreversibly collapses, leading to the formation of a mixed metal oxide matrix predominantly composed of nanocrystalline ZnO embedded in an amorphous alumina-rich phase. This transformation aligns with the XRD data, where the disappearance of LDH reflections and the emergence of ZnO diffraction peaks confirm the structural breakdown. Despite the collapse, the amorphous oxide retains sufficient short-range order to enable the reconstruction of the LDH phase upon hydration in the presence of suitable anions, provided that further crystallization into spinel does not occur.^30^

This controlled thermal treatment is particularly effective for replacing carbonate with functional anions, such as DBM. Calcination at approximately 460 °C efficiently removes the carbonate species while preserving the reactive amorphous oxide framework necessary for the memory effect. Finally, the fourth stage, observed above 500 °C, is associated with the release of residual carbonates and the crystallization of stable mixed oxide phases, where the memory effect is permanently lost.^18^

When compared to undoped Zn/Al LDHs reported in the literature, the Eu^3+^-doped sample exhibits weight loss events shifted to slightly lower temperatures, indicating that europium incorporation reduces the thermal stability of the lamellar structure. This effect likely arises from local distortions induced by the larger ionic radius and the electronic configuration of Eu^3+^ within the brucite-like layers.

The structural reconstruction of the calcined ZAE5-460 sample was evaluated through rehydration in the presence of dibenzoylmethane (DBM) as a guest anion (Figure 5). The XRD pattern of the sample after 1 h of rehydration (ZAE5-DBM-60 min) shows the partial recovery of the lamellar structure, as evidenced by the appearance of a basal reflection at 11.9° (2θ), which corresponds to a basal spacing of approximately 0 = 0.743 nm. This value is consistent with the intercalation of DBM anions oriented parallel to the layers, as reported for aromatic dicarbonyl compounds in LDHs.^12,31^

**Figure 5:**
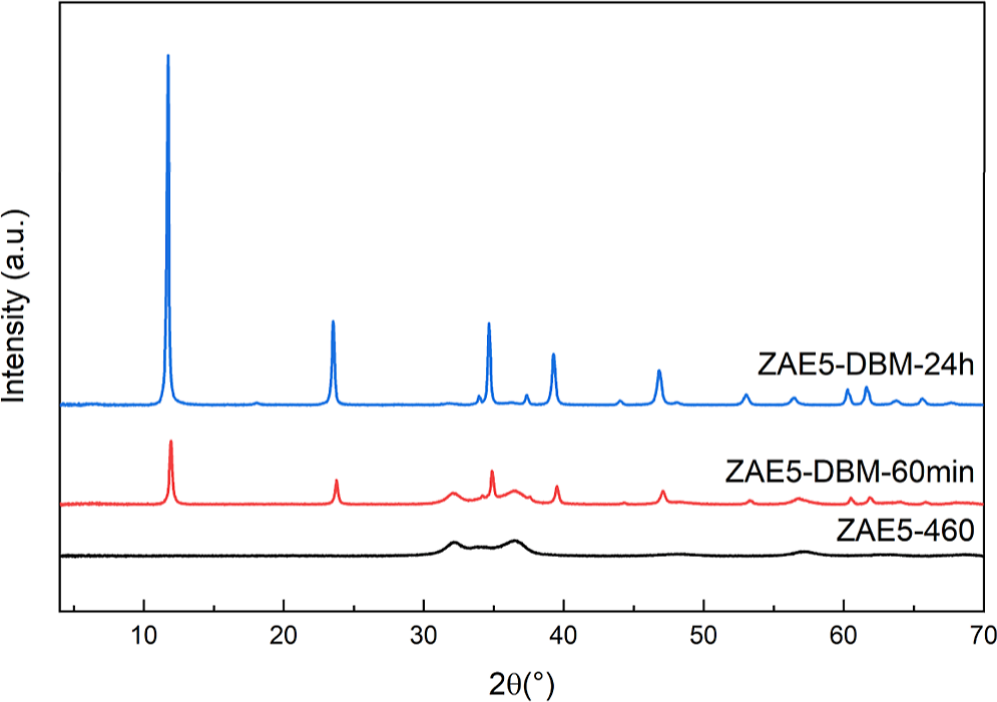
PXRD patterns of the calcined LDH (ZAE5-460) after rehydration in DBM solution for different times (60 min and 24 h). The reappearance of the lamellar structure and the evolution of DBM intercalation are shown. Residual ZnO reflections are noted in the 60 min sample.

However, the presence of residual diffraction peaks at 31.7°, 34.4°, and 36.2° (2θ) corresponding to the (100), (002), and (101) planes of ZnO wurtzite (JCPDS 36-1451) indicates that the reconstruction process is incomplete after 1 h (Figure S2). This suggests that a portion of the oxide matrix remains unconverted, either due to kinetic limitations in the hydration process or the presence of ZnO domains that are structurally stabilized and no longer reactive toward rehydration.

After 24 h of rehydration (ZAE5-DBM-24 h), the basal reflection at 11.9° (2θ) becomes significantly more intense, narrower, and sharper, indicating a higher degree of long-range order along the *c*-axis (Figure S1). Crucially, the ZnO-related reflections completely vanish, confirming the full reconstruction of the LDH structure and the successful intercalation of DBM anions. This result demonstrates that the amorphous mixed-metal oxide matrix produced by thermal decomposition at 460 °C retains the chemical memory necessary to regenerate the lamellar architecture when exposed to appropriate conditions.

It is worth noting that the final interlayer distance remains lower than typical carbonate-intercalated LDHs (*d*_003_ = 0.756 nm) but is fully consistent with the presence of DBM (Figure S3), which adopts a flat orientation between the layers due to its aromatic nature and strong coordination via β-diketone functional groups. Unlike systems where DBM is part of a preformed Eu^3+^ complex containing three DBM ligands and a bulky auxiliary such as bathophenanthrolinedisulfonic acid (as in the EuL_3_ complex reported by Sarakha et al.^21^), the present system involves direct intercalation of DBM during the rehydration process, without additional spacers or coordinating ligands. In that study, the LDH intercalated with Eu­(DBM)_3_-BPS exhibited an interlayer distance of 2.10 nm nearly three times greater than the 0.743 nm observed here which reflects the significantly larger molecular volume of the Eu­(DBM)_3_–BPS complex. In contrast, the compact structure obtained in our system suggests that the DBM is incorporated in a more planar and space-efficient configuration. This observation aligns with previous studies on organic–inorganic hybrid LDHs and further confirms the versatility of the memory effect in enabling postsynthetic functionalization.^17^

Elemental analysis by ICP-OES (Table S1) confirms that the cationic composition of the LDH matrix remains stable throughout the synthesis, calcination, and rehydration. All samples exhibit Zn/(Al + Eu) ratios close to 2, consistent with the expected brucite-like LDH structure. The measured Al^3+^/Eu^3+^ molar ratio for the ZAE5-CO_3_ sample was approximately 0.94:0.03, closely matching the nominal composition used in the synthesis (0.95:0.05). After calcination, the Eu content increases slightly to 0.05, which may result from improved detection sensitivity, redistribution of ions upon thermal treatment, or minor experimental variations typically observed at low doping levels. These results confirm the successful incorporation of Eu^3+^ into the LDH lattice and its retention during memory-effect-driven reconstruction.

The CHN (Table S1) results reveal the expected removal of carbonate and structural water upon calcination, evidenced by a sharp decrease in carbon (from 7.67 to 0.69 wt %) and hydrogen (from 2.73 to 0.57 wt %). This aligns with the thermal decomposition events identified in the TG/DTG analysis and the collapse of the lamellar structure seen in the XRD patterns.

Upon rehydration with DBM, the increase in carbon (2.27 wt %) and hydrogen (2.98 wt %) confirms the incorporation of the organic ligand into the interlayer space. Additionally, the significant rise in nitrogen content (from 0.03 to 0.40 wt %) suggests the cointercalation or adsorption of nitrogen-containing species, likely residual nitrate from the synthesis medium. This is corroborated by FTIR data, where bands associated with nitrate persist alongside the characteristic vibrations of the DBM ligand, indicating that the reconstruction of the layered structure involves not only DBM but also minor amounts of inorganic species.

This elemental profile reinforces the XRD evidence of the memory effect and supports the successful intercalation of DBM, while also highlighting the presence of secondary species influencing the final material composition.

The FTIR spectra (Figure 6) provide further insights into the structural evolution of the samples across the different synthesis steps. For the ZAE5-CO_3_ sample, the spectrum exhibits the characteristic features of LDHs. A broad band around 3400 cm^–1^ corresponds to the stretching vibrations of hydroxyl groups (O–H) associated both with the brucite-like layers and adsorbed water.^32,33^ The bending mode of interlayer water appears near 1630 cm^–1^. A strong, sharp absorption at 1360 cm^–1^ is attributed to the antisymmetric stretching (ν_3_) of interlayer carbonate ions, consistent with the XRD results showing the basal reflection of the lamellar structure at 11.3° (JCPDS 0048-1023).^34^

**Figure 6:**
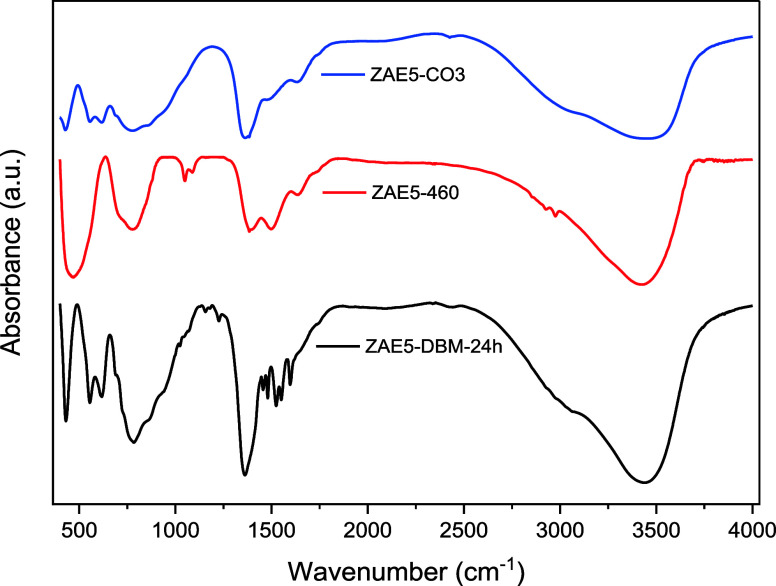
FTIR spectra of ZAE5-CO_3_, ZAE5-460, and ZAE5-DBM-24 h. The bands associated with hydroxyl stretching, carbonate vibrations, and DBM functional groups are indicated, confirming the successful removal and subsequent intercalation of DBM.

Upon calcination at 460 °C (ZAE5-460), the FTIR spectrum shows a drastic reduction in the carbonate band at 1360 cm^–1^, confirming the complete removal of interlayer carbonate, in line with the TG data that indicated total decomposition at around 441 °C. Similarly, the band associated with structural hydroxyls (∼3400 cm^–1^) is significantly diminished, confirming the dehydroxylation of the LDH layers. However, a residual O–H stretching band persists in the spectrum. This feature arises from the readsorption of atmospheric moisture onto the high-surface-area mixed oxide at room temperature during the FTIR measurement, a behavior typical of LDH-derived oxides.

This observation is coherent with the XRD pattern of ZAE5-460, which shows the disappearance of lamellar peaks but retains reflections of ZnO and amorphous oxides.

After rehydration and intercalation of DBM (ZAE5-DBM-24 h), the FTIR spectrum undergoes marked changes. The O–H stretching band at 3400 cm^–1^ is partially restored, indicating the reconstruction of the hydroxide layers via the memory effect. The complete disappearance of ZnO reflections in the XRD data confirms this structural recovery. The carbonate band at 1360 cm^–1^ is absent, confirming that the interlayer carbonate has been successfully replaced by the organic anion. New bands emerge in the range 1600–1450 cm^–1^, assigned to C=C stretching from the aromatic rings of DBM. A prominent band at 1530 cm^–1^ corresponds to the ν­(C=O) of the β-diketonate group coordinated to the metal centers.^34,35^ These vibrational signatures confirm the successful intercalation of DBM into the LDH matrix, corroborating the elemental analysis results that showed a significant increase in the C, H, and N contents.

The restoration of the lamellar structure with DBM intercalation not only is confirmed by XRD and FTIR but also plays a crucial role in the luminescence behavior discussed later. The re-establishment of a symmetric layered environment reduces nonradiative deactivation pathways, enhancing the Eu^3+^ emission intensity compared to the oxide phase.

The SEM micrographs provide valuable insights into the morphological evolution of the ZAE5-CO_3_-based materials during the processes of calcination and subsequent reconstruction via the memory effect with DBM intercalation (Figure 7).

**Figure 7:**
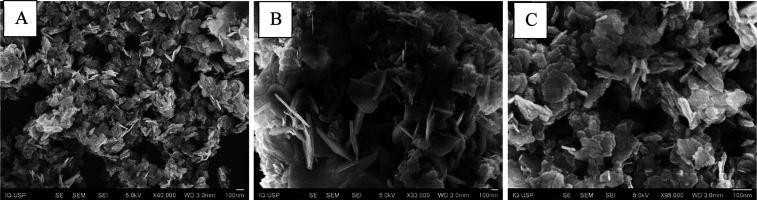
SEM images of (A) ZnAlEu_5_-CO_3_, (B) ZAE5-460, and (C) ZAE5-DBM-24 h. The morphological differences associated with the lamellar structure, oxide phase, and rehydrated hybrid material are shown.

The ZAE5-CO_3_ sample (Figure 7A) exhibits a typical lamellar morphology, characteristic of LDHs, with well-defined, thin, plate-like structures. These platelets are loosely stacked, forming aggregated clusters with a high degree of porosity. This morphology aligns with the XRD data, which confirms the presence of the hydrotalcite-like phase and is consistent with the strong OH and carbonate vibration bands observed in FTIR. The chemical composition from ICP and CHN also supports this structure, with high hydrogen and carbon contents attributed to interlayer water and carbonate anions.

After calcination at 460 °C (Figure 7B), a morphological transformation is observed. The lamellar structure collapses, giving rise to a disordered, porous oxide framework composed of irregularly shaped particles. Despite the thermal treatment, the micrograph reveals that the material retains a certain degree of mesoporosity, which is crucial for enabling the memory effect. This observation correlates with the TG results, where decomposition of carbonate and dehydroxylation lead to the amorphous mixed metal oxides (ZnO). The presence of surface hydroxyl groups, detected in the FTIR as residual OH stretching bands, suggests rehydration from atmospheric moisture postcalcination, even though the lamellar order is no longer present.

Following the memory effect reconstruction with DBM intercalation (Figure 7C), the material recovers a pseudolamellar morphology. The micrograph shows the formation of thinner, more disordered nanosheets compared to the regular material. This reconstructed architecture, while not as ordered as the original LDH, demonstrates successful reintegration of layered domains, consistent with the XRD patterns showing the return of basal reflections at lower angles. Additionally, the smoother sheet-like features indicate the successful incorporation of DBM anions, which is supported by the significant increase in C, H, and N contents from CHN analysis, and by the FTIR bands associated with aromatic and β-diketone groups.

The photoluminescence properties were investigated through excitation and emission spectra recorded at room temperature for all samples (Figure 8).

**Figure 8:**
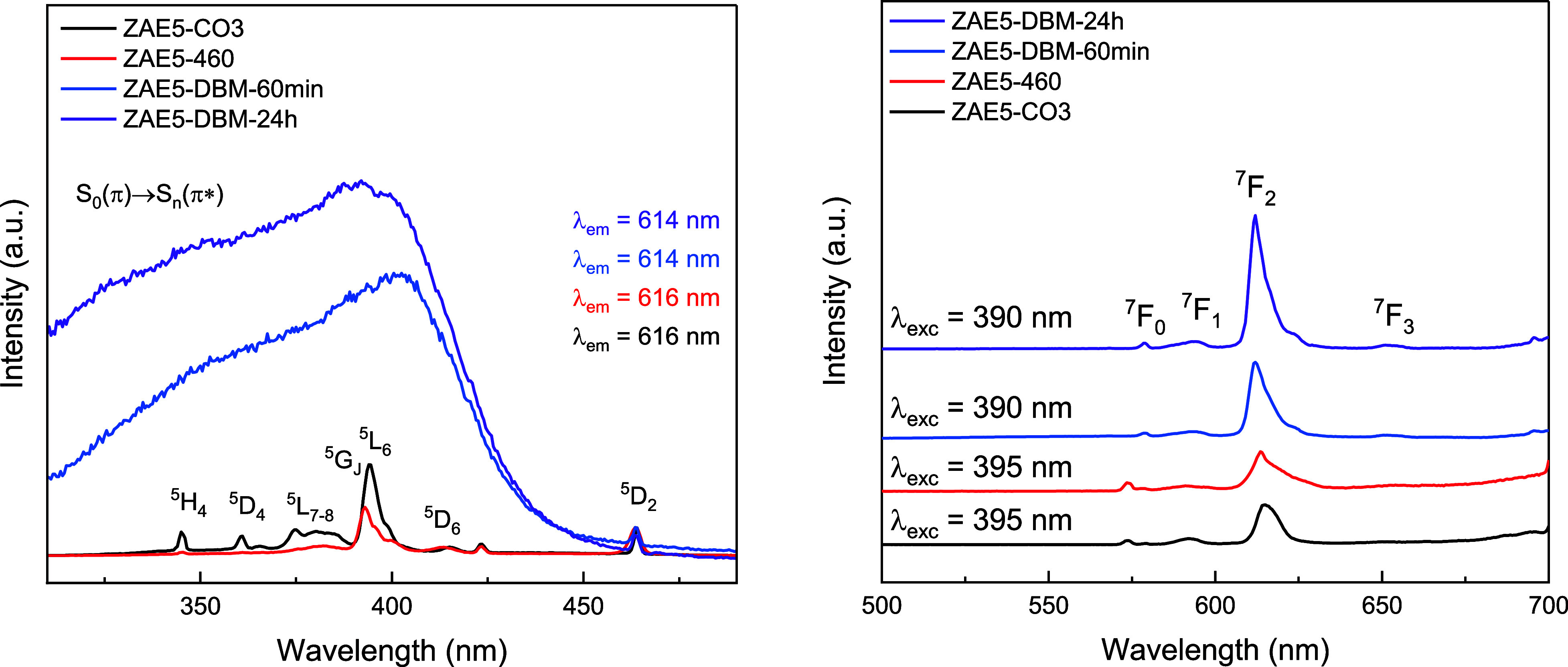
(Left) Normalized excitation spectra recorded at 300 K, monitoring the Eu^3+^ ^5^D_0_ → ^7^F_2_ emission at 614 nm for ZAE5-CO_3_, ZAE5-460, and the rehydrated samples ZAE5-DBM-60 min and ZAE5-DBM-24 h. (Right) Emission spectra under excitation at 390 nm (DBM band) for the rehydrated samples and 395 nm (Eu^3+^ direct excitation) for ZAE5-CO_3_ and ZAE5-460. The emergence of the antenna effect is indicated by enhanced emission and the presence of ligand excitation.

The excitation spectra, monitored for the characteristic red emission of Eu^3+^ (^5^D_0_ → ^7^F_2_), are shown in Figure 8 (left). The regular sample (ZAE-CO_3_), which contains intercalated carbonate, exhibits only the sharp intra transitions of Eu^3+^, including the hypersensitive ^7^F_0_ → ^5^L_6_ and ^7^F_0_ → ^5^D_2_ bands. No broad absorption bands related to ligand-centered excitation are observed, indicating the absence of antenna ligands capable of sensitizing Eu^3+^ luminescence.

A similar behavior is observed in the thermally treated ZAE5-460 sample, which also exhibits exclusively the characteristic Eu^3+^ transitions. This confirms that the calcination process at 460 °C effectively removes the carbonate and any residual organic species, resulting in a disordered mixed oxide phase, where Eu^3+^ ions remain isolated, with no coordination to sensitizing ligands. This is further evidence by the excitation spectrum, which closely matches that of Eu_2_O_3_, showing only characteristic 4f–4f transitions.^36,37^

Upon rehydration of ZAE5-460 in the presence of DBM via the memory effect, significant changes are observed in the excitation spectra of the resulting ZAE5-DBM-60 min and ZAE5-DBM-24 h samples. In both cases, in addition to the Eu^3+^ 4f–4f transitions, a broad absorption band emerges in the 300–400 nm region, attributed to the S_0_(π) → S_*n*_(π*) transitions of the coordinated DBM ligands.^21^ This observation is consistent with previous reports on Eu^3+^ β-diketonate complexes, where DBM acts as a sensitizer through the well-known antenna effect.^38,39^

The presence of this band confirms the successful intercalation of DBM anions into the LDH structure via the memory effect, leading to reconstitution of the layered structure and coordination of Eu^3+^ ions by the organic ligand. Notably, this band overlaps with the 7F_0_ → 5L_6_ transition of Eu^3+^, suggesting a competitive and efficient ligand-to-metal energy transfer pathway.

The intensity of the DBM band is significantly higher in the ZAE5-DBM-24 h sample compared with the ZAE5-DBM-60 min sample. This indicates that longer exposure to the DBM solution favors more extensive intercalation and stronger coordination between DBM ligands and Eu^3+^ ions, resulting in enhanced energy transfer efficiency. Consequently, the red emission intensity under ligand excitation is also markedly increased, demonstrating the role of DBM in modulating the luminescence behavior of the hybrid LDH system.

In contrast, the ZAE-CO_3_ and ZAE5-460 samples show no evidence of ligand-based excitation, confirming that the antenna effect and the associated luminescence enhancement are only activated upon DBM intercalation. These results clearly demonstrate the effectiveness of the memory effect as a strategy to functionalize Eu^3+^-doped LDHs with luminescent organic anions.

The emission spectra (right) recorded at room temperature for all samples exhibit the characteristic Eu^3+^ transitions from the ^5^D_0_ excited state to the ^7^F_*J*_ (*J* = 0, 1, 2, and 3) manifold. In all samples, the most intense transition corresponds to the hypersensitive ^5^D_0_ → ^7^F_0_ electric dipole transition (∼614–616 nm), which is highly sensitive to the local symmetry of the Eu^3+^ coordination environment. The magnetic dipole transition (^5^D_0_ → ^7^F_0_), typically insensitive to local symmetry, is present but less intense, while the ^5^D_0_ → ^7^F_0_ transition appears with varying intensity, indicating differences in the symmetry of the Eu^3+^ sites among the samples.

A comparison of the emission intensities reveals a substantial enhancement for the DBM-intercalated samples (ZAE5-DBM-60 min and ZAE5-DBM-24 h) relative to that of the ZAE-CO_3_ and calcined (ZAE5-460) samples. This enhancement correlates with the appearance of the broad S_0_(π) → S_*n*_(π*) excitation band around 390 nm in the DBM-containing samples absent in the others demonstrating efficient energy transfer from the DBM ligand to the Eu^3+^ ion via the antenna effect.

Beyond intensity enhancement, distinct spectral shifts are observed in the peak positions of the Eu^3+^ transitions (Table 1). Specifically, the ^5^D_0_ → ^7^F_0_ transition shifts from 573.6 nm in ZAE-CO_3_ and ZAE5-460 to 578.5 nm in the DBM-intercalated samples. Conversely, the ^5^D_0_ → ^7^F_2_ transition exhibits a slight blueshift from ∼616.2 to 616.5 nm (in the samples without DBM) to ∼614.3–614.5 nm (in the DBM-containing samples).

**Table 1:** Photoluminescence Parameters of LDH Samples: Transition Energies, Asymmetry Ratio (Ω_2_), and Quantum Yield

samples	^5^D_0_ → ^7^F_0_	^5^D_0_ → ^7^F_2_	Ω_2_	η (%)
ZAE-CO_3_	573.56 nm	616.24 nm	15.1	0.06
ZAE-460	573.66 nm	616.51 nm	20.0	0.16
ZAE-DBM-60 min	578.55 nm	614.47 nm	27.2	11.89
ZAE-DBM-24 h	578.51 nm	614.28 nm	30.1	13.57
Sarakha et al.^21^				8.3; 7.0

These shifts reflect changes in the ligand field strength and the symmetry around the Eu^3+^ ion.^40^ The redshift of the ^5^D_0_ → ^7^F_0_ transition is indicative of an increased nephelauxetic effect, which suggests greater delocalization of the electron density and enhanced covalency in the europium ligand bonds upon DBM coordination. In parallel, the slight blueshift observed in the ^5^D_0_ → ^7^F_0_ transition is associated with a reduction in local symmetry, a phenomenon reported in the literature for systems where Eu ligand bond elongation and lower point group symmetry lead to similar spectral behavior.^40–42^

To gain deeper insight into these symmetry changes, the Judd–Ofelt asymmetry parameter Ω_2_ was calculated from the emission spectra. This parameter quantifies the relative strength of the ^5^D_0_ → ^7^F_2_ electric dipole transition in relation to the ^5^D_0_ → ^7^F_1_ magnetic dipole transition based on their integrated intensities and barycenter positions. The calculation follows the expression:

1 $$\Omega_{2}=\frac{S_{2}}{S_{1}}\cdot \frac{9n{\left(n^{2}+2\right)}^{2}}{32\pi^{2}n^{3}e^{2}U_{2}^{2}D_{MD}}$$

where *S*_2_ and *S*_1_ are the integrated intensities of the ^5^D_0_ → ^7^F_2_ and ^5^D_0_ → ^7^F_1_ transitions, respectively, *n* is the refractive index of the matrix (assumed to be 1.5), *e* is the elementary charge, *U*_2_ is the reduced matrix element (tabulated), and *D*_MD_ is the dipole strength of the magnetic dipole transition, typically 9.6 × 10^–6^ debye^2^.^12^

This increase in the structural asymmetry correlates with the observed luminescence enhancement. While the ZAE5-CO_3_ and ZAE5-460 samples display modest quantum yields, the intercalated materials achieve significantly higher efficiencies (Table 1), confirming the dual role of DBM: as a sensitizing ligand via the antenna effect and as a structural modifier that tunes the local environment around Eu^3+^.

A comparison with the previous literature highlights the significance of this improvement. In the study by Sarakha et al.,^21^ EuL_3_ complexes with DBM ligands exhibited a high quantum yield of 46.2% in solution, but this efficiency decreased sharply to 8.3% and 7.0% when intercalated into Zn_2_Al–LDH and Zn_4_Al–LDH hosts, respectively, due to nonradiative deactivation via hydroxyl groups within the layers. In contrast, the DBM-intercalated samples obtained via memory-effect reconstruction in this study maintain superior emission performance. This difference is likely related to the more effective DBM coordination achieved during the rehydration step as well as the reduced presence of luminescence quenchers in the reconstructed host matrix.

These findings demonstrate that the memory effect provides a robust and versatile strategy for the functionalization of Eu^3+^-doped LDHs with organic ligands, enabling precise tuning of photophysical behavior through control over both the structural and chemical environments.

## 4 Conclusions

In summary, we demonstrated the successful functionalization of Eu^3+^-doped ZnAl LDHs via the memory effect, enabling the incorporation of DBM as an organic luminescent ligand. The LDH intercalated with carbonate exhibited only the characteristic transitions of Eu^3+^, with no evidence of ligand-to-metal energy transfer. Upon calcination, the layered structure assumed a disoriented form, leading to the formation of an amorphous mixed oxide still containing isolated Eu^3+^ ions, without any remarkable luminescence.

Rehydration of calcined LDH in DBM solution restored the layered structure and enabled the intercalation of DBM anions. This process drastically altered the photophysical properties of the material. The appearance of a broad S_0_(π) → S_*n*_(π*) absorption band in the excitation spectra and the significant enhancement of red emission intensity are clear evidence of an efficient antenna effect promoted by the DBM ligands. Moreover, spectral shifts observed in the emission profiles confirm modifications in the ligand field surrounding the Eu^3+^ ions, reflecting stronger covalent interactions and changes in symmetry induced by DBM coordination.

This interpretation is further supported by the calculated asymmetry ratio (Ω_2_), which increases notably upon DBM intercalation—from 15.1 (ZAE5-CO_3_) and 20.0 (ZAE5-460) to 27.2 and 30.1 for the DBM-functionalized samples after 60 min and 24 h of rehydration, respectively. These elevated values are indicative of a more distorted coordination environment around Eu^3+^, consistent with enhanced ligand–metal interactions and reduced local symmetry. Such structural modifications align closely with the observed quantum efficiency values, which rise from just 0.06% and 0.16% in the nonfunctionalized samples to 11.89% and 13.57% following DBM intercalation.

Altogether, these findings not only confirm the successful activation of the antenna effect but also establish the memory effect as a unique and effective route for intercalating bulky β-diketonate ligands such as DBM, which cannot be incorporated by conventional synthesis methods. Additionally, the use of extended characterization such as SEM, FTIR, ICP-OES, CHN, and Judd–Ofelt analysis allowed a deeper understanding of the intermediate and final stages of the rehydration process. These comprehensive insights and the markedly improved luminescent behavior demonstrate a substantial advancement over previous studies and reinforce the potential of memory-effect-assisted strategies in designing high-performance luminescent LDH-based hybrid materials for optoelectronic, sensing, and photonic applications.

## Supplementary Material



## Notes

X-ray diffraction patterns for the samples ZAE-450, ZAE-DMB-60 min, and ZAE-DMB-24 h and chemical analysis of LDHs (PDF)

## References

[1] Lisevski C. I., Morais A. F., Aguero N. F., Teixeira A. C., Moreira
Ribeiro F. W., Correra T. C., Guide Nunes da Silva I., Mustafa D. (2024). Vitamin B_3_ Intercalated in Layered Double
Hydroxides: A Drug Delivery System for Metabolic Regulation. ACS Omega.

[2] Pastor A., Chen C., de Miguel G., Martín F., Cruz-Yusta M., O’Hare D., Pavlovic I., Sánchez L. (2023). Facile Synthesis
of Visible-Responsive Photocatalytic Eu-Doped Layered Double Hydroxide
for Selective Removal of NOx Pollutant. Chem.
Eng. J..

[3] Oliva M. D. L. Á., Chen C., de Miguel G., O’Hare D., Pavlovic I., Sánchez L., Pastor A. (2024). Europium Insertion into MgAl Hydrotalcite-like Compound
to Promote the Photocatalytic Oxidation of Nitrogen Oxides. Chemosphere.

[4] Morais A. F., Silva I. G. N. N., Sree S. P., de Melo F. M., Brabants G., Brito H. F., Martens J. A., Toma H. E., Kirschhock C. E. A. A., Breynaert E., Mustafa D. (2017). Hierarchical Self-Supported ZnAlEu
LDH Nanotubes Hosting Luminescent CdTe Quantum Dots. Chem. Commun..

[5] Morais A. F., Silva I. G. N., Ferreira B. J., Teixeira A. C., Sree S. P., Terraschke H., Garcia F. A., Breynaert E., Mustafa D. (2023). Eu3+ Doped ZnAl Layered Double Hydroxides as Calibrationless,
Fluorescent Sensors for Carbonate. Chem. Commun..

[6] Gunawan P., Xu R. (2009). Lanthanide-Doped Layered Double Hydroxides Intercalated with Sensitizing
Anions: Efficient Energy Transfer between Host and Guest Layers. J. Phys. Chem. C.

[7] Zhao Y., Li J. G., Fang F., Chu N., Ma H., Yang X. (2012). Structure and Luminescence Behaviour of As-Synthesized, Calcined,
and Restored MgAlEu-LDH with High Crystallinity. Dalton Trans..

[8] Mustafa D., Biggemann D., Wu J., Coffer J. L., Tessler L. R. (2007). Structural
Characterization of ZnO/Er_2_O_3_ Core/Shell Nanowires. Superlattices Microstruct..

[9] Morais A. F., Silva I. G. N., Lima B. C., Garcia F. A., Mustafa D. (2020). Coordination
of Eu3+ Activators in ZnAlEu Layered Double Hydroxides Intercalated
by Isophthalate and Nitrilotriacetate. ACS Omega.

[10] Teixeira A. C., Nunes Silva I. G., Morais A. F., Mustafa D. (2022). Structural and Optical
Properties of Pillared Eu^3+^-Containing Layered Double Hydroxides
Intercalated by 2- to 12-Carbon Aliphatic Dicarboxylates. J. Rare Earths.

[11] Gawryszewska P., Miller L. W., Valente A. J. M. (2023). Editorial: Hot
Topic: Luminescence
in Rare Earth Coordination Compounds. Front.
Chem..

[12] Binnemans K. (2015). Interpretation
of Europium­(III) Spectra. Coord. Chem. Rev..

[13] Zhu Q., Liu J., Li X., Sun X., Li J.-G. (2019). Grafting Organic
Antenna onto Rare Earth Hydroxynitrate Nanosheets for Excitation-Dependent
and Greatly Enhanced Photoluminescence by Multi-Modal Energy Transfer. Appl. Surf. Sci..

[14] Morais A. F., Machado F. O., Teixeira A. C., Silva I. G. N., Breynaert E., Mustafa D. (2019). Enhanced Luminescence
in ZnAlEu Layered Double Hydroxides
with Interlamellar Carboxylate and β-Diketone Ligands. J. Alloys Compd..

[15] Nanclares D., Morais A. F., Calaça T., Silva I. G. N., Mustafa D. (2021). A Class of
Novel Luminescent Layered Double Hydroxide Nanotubes. RSC Adv..

[16] Matsuda K., Okuda A., Iio N., Tarutani N., Katagiri K., Inumaru K. (2024). Chemical and Structural Transformations of M-Al-CO3
Layered Double Hydroxides (M = Mg, Zn, or Co, M/Al = 2) at Elevated
Temperatures: Quantitative Descriptions and Effect of Divalent Cations. Inorg. Chem..

[17] Teixeira A., Morais A., Silva I., Breynaert E., Mustafa D. (2019). Luminescent Layered Double Hydroxides Intercalated
with an Anionic Photosensitizer via the Memory Effect. Crystals.

[18] Kowalik P., Konkol M., Kondracka M., Próchniak W., Bicki R., Wiercioch P. (2013). Memory Effect
of the CuZnAl-LDH Derived
Catalyst Precursor - In Situ XRD Studies. Appl.
Catal., A.

[19] Pattappan D., Kapoor S., Islam S. S., Lai Y.-T. (2023). Layered Double Hydroxides
for Regulating Phosphate in Water to Achieve Long-Term Nutritional
Management. ACS Omega.

[20] Nemček L., Hagarová I., Matúš P. (2024). Layered Double
Hydroxides as Next-Generation
Adsorbents for the Removal of Selenium from Water. Appl. Sci..

[21] Sarakha L., Forano C., Boutinaud P. (2009). Intercalation
of Luminescent Europium­(III)
Complexes in Layered Double Hydroxides. Opt.
Mater..

[22] Silva I. G. N., Mustafa D., Felinto M. C. F. C., Faustino W. M., Teotonio E. E. S., Malta O. L., Brito H. F. (2015). Low Temperature
Synthesis of Luminescent
RE_2_O_3_:Eu^3+^ Nanomaterials Using Trimellitic
Acid Precursors. J. Braz. Chem. Soc..

[23] Huang P.-P., Cao C.-Y., Wei F., Sun Y.-B., Song W.-G. (2015). MgAl Layered
Double Hydroxides with Chloride and Carbonate Ions as Interlayer Anions
for Removal of Arsenic and Fluoride Ions in Water. RSC Adv..

[24] Parida K., Mohapatra L., Baliarsingh N. (2012). Effect of Co2+ Substitution in the
Framework of Carbonate Intercalated Cu/Cr LDH on Structural, Electronic,
Optical, and Photocatalytic Properties. J. Phys.
Chem. C.

[25] Magdy
Abdo D., Nasr El-Shazly A., Adel Hamza M. (2024). Effect of
Acidic and Basic Leachants on the Synthesis of ZnO Nanoparticles from
Egyptian Zinc Ore, Their Optical/Surface Characteristics and Photocatalytic
Performance. Mater. Lett..

[26] Zhao X., Wang L., Xu X., Lei X., Xu S., Zhang F. (2012). Fabrication and Photocatalytic Properties of Novel ZnO/ZnAl_2_O_4_ Nanocomposite with ZnAl_2_O_4_ Dispersed
inside ZnO Network. AIChE J..

[27] Santos R. M. M., Tronto J., Briois V., Santilli C. V. (2017). Thermal Decomposition
and Recovery Properties of ZnAl–CO_3_ Layered Double
Hydroxide for Anionic Dye Adsorption: Insight into the Aggregative
Nucleation and Growth Mechanism of the LDH Memory Effect. J. Mater. Chem. A.

[28] Liu S.-T., Zhang P.-P., Yan K., Zhang Y.-H., Ye Y., Chen X.-G. (2015). Sb-intercalated Layered Double Hydroxides–Poly­(Vinyl
Chloride) Nanocomposites: Preparation, Characterization, and Thermal
Stability. J. Appl. Polym. Sci..

[29] Inayat A., Makky A., Giraldo J., Kuhnt A., Busse C., Schwieger W. (2014). Thermally Induced Growth of ZnO Nanocrystals on Mixed
Metal Oxide Surfaces. Chem. - Eur. J..

[30] Wong F., Buchheit R. G. (2004). Utilizing the Structural
Memory Effect of Layered Double
Hydroxides for Sensing Water Uptake in Organic Coatings. Prog. Org. Coat..

[31] Mech A., Karbowiak M., Görller-Walrand C., Van Deun R. (2008). The Luminescence
Properties of Three Tetrakis Dibenzoylmethane Europium­(III) Complexes
with Different Counter Ions. J. Alloys Compd..

[32] Yasaei M., Khakbiz M., Ghasemi E., Zamanian A. (2019). Synthesis and Characterization
of ZnAl-NO_3_ (-CO_3_) Layered Double Hydroxide:
A Novel Structure for Intercalation and Release of Simvastatin. Appl. Surf. Sci..

[33] Salak A. N., Tedim J., Kuznetsova A. I., Ribeiro J. L., Vieira L. G., Zheludkevich M. L., Ferreira M. G. S. (2012). Comparative X-Ray Diffraction and
Infrared Spectroscopy Study of Zn-Al Layered Double Hydroxides: Vanadate
vs Nitrate. Chem. Phys..

[34] Tanaka T., Kameshima Y., Nishimoto S., Miyake M. (2012). Determination of Carbonate
Ion Contents in Layered Double Hydroxides by FTIR Spectrometry. Anal. Methods.

[35] Wall F. T., Claussen W. F. (1939). Infrared Absorption Spectra of Some Carboxylic Acids
and of Dibenzoylmethane and Related Molecules. J. Am. Chem. Soc..

[36] Worsley M. A., Ilsemann J., Gesing Th. M., Zielasek V., Nelson A. J., Ferreira R. A. S., Carlos L. D., Gash A. E., Bäumer M. (2019). Chlorine-Free,
Monolithic Lanthanide Series Rare Earth Oxide Aerogels via Epoxide-Assisted
Sol-Gel Method. J. Sol-Gel Sci. Technol..

[37] Xin Y., Wang Z., Qi Y., Zhang Z., Zhang S. (2010). Synthesis
of Rare Earth (Pr, Nd, Sm, Eu and Gd) Hydroxide and Oxide Nanorods
(Nanobundles) by a Widely Applicable Precipitation Route. J. Alloys Compd..

[38] Brito H. F., Carvalho C. A. A., Malta O. L., Passos J. J., Menezes J. F. S., Sinisterra R. D. (1999). Spectroscopic Study of the Inclusion
Compound of β-Cyclodextrin and Tris­(Dibenzoylmethane)­Europium­(III)
Dihydrate. Spectrochim. Acta, Part A.

[39] Meng Q., Boutinaud P., Zhang H., Mahiou R. (2007). Luminescence Properties
of Eu3+ β-Diketonates Incorporated in Cubic Mesoporous Silica. J. Lumin..

[40] Jørgensen C. K., Reisfeld R. (1983). Judd-Ofelt Parameters and Chemical Bonding. J. Less-Common Met..

[41] Albin M., Horrocks W. D. (1985). Europium­(III) Luminescence Excitation
Spectroscopy.
Quantitive Correlation between the Total Charge on the Ligands and
the 7F0.Fwdarw. 5D0 Transition Frequency in Europium­(III) Complexes. Inorg. Chem..

[42] Yi X., Sun J., Jiang X.-F., Li Y., Xu Q.-H., Zhang Q., Ye S. (2016). Variations in the ^5^D_0_ → ^7^F_0–4_ Transitions of Eu ^3+^ and White
Light Emissions in Ag–Eu Exchanged Zeolite-Y. RSC Adv..

