# Supplementary material for: Efficient Energy Transfer in Eu3+-Doped Layered Double Hydroxides with β‑Diketonate Anions Obtained by the Memory Effect
Source: ACS Omega. 2025 Sep 17;10(38):44137–46. doi: 10.1021/acsomega.5c05499 (PMC12489650; doi:10.1021/acsomega.5c05499)
Supplement: Supplementary file 1 [file ao5c05499_si_001.pdf]

Supporting information

Efficient energy transfer in Eu^{3+} doped Layered Double Hydroxides with β-diketonate anions obtained by Memory Effect

Alexandre Candido Teixeira, Natan Felipe Netzlaff Fachini, Henrique Kenzo Carvalho Kakinami and Danilo Mustafa*

Instituto de Física da Universidade de São Paulo, 05508-090 São Paulo, SP, Brazil;

* Email: dmustafa@if.usp.br

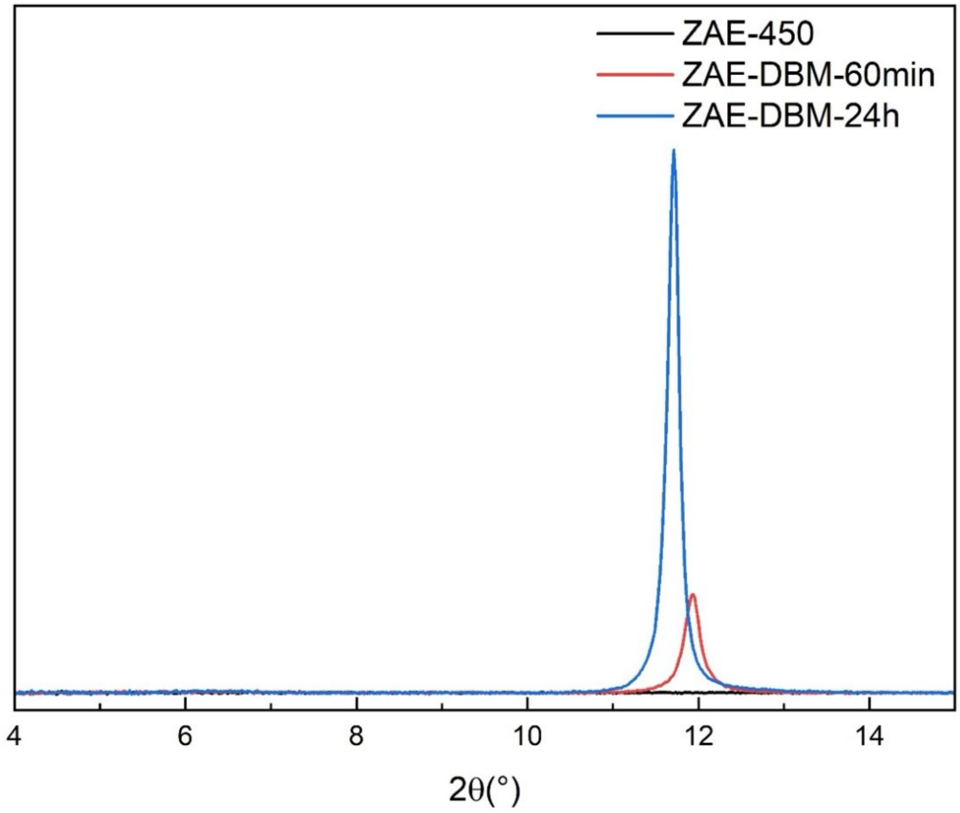

Figure S1. Expanded view of the XRD patterns in the low-angle region (4–15°) highlighting the (003) basal reflection. The shift of this peak from 11.72° in the ZAE5–CO_3 sample to 11.90° in the DBM-intercalated samples confirms the successful reconstruction of the LDH structure via the memory effect, with an expanded interlayer spacing associated with DBM incorporation.

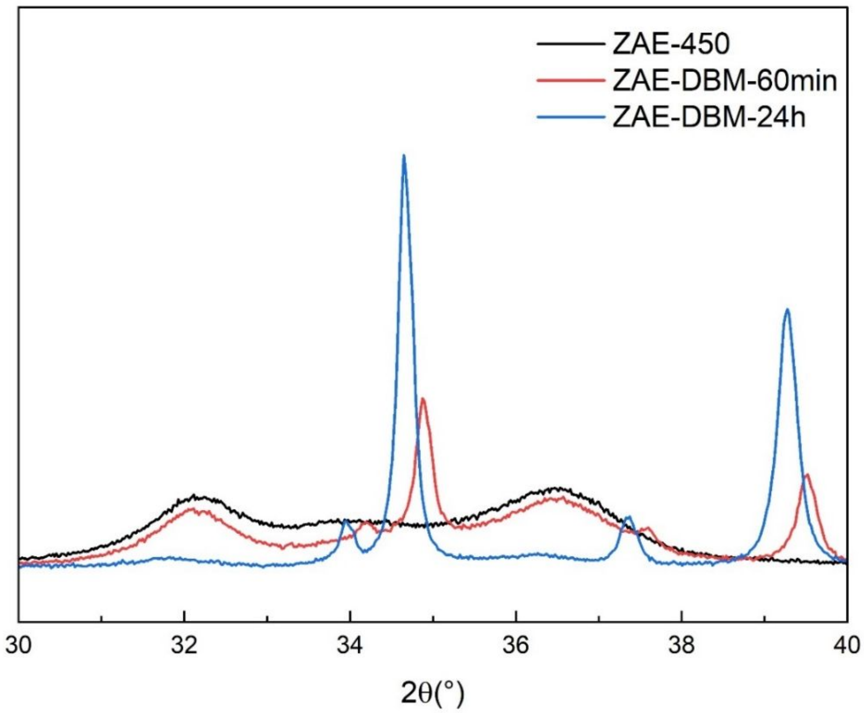

Figure S2. Magnified XRD patterns in the 2θ range of 30-40°, emphasizing the ZnO (101) reflection. The presence of this peak in the calcined sample (ZAE5–460) indicates the formation of ZnO after thermal decomposition. Its gradual suppression in the DBM-rehydrated samples demonstrates the progressive restoration of the layered double hydroxide structure during the memory effect process.

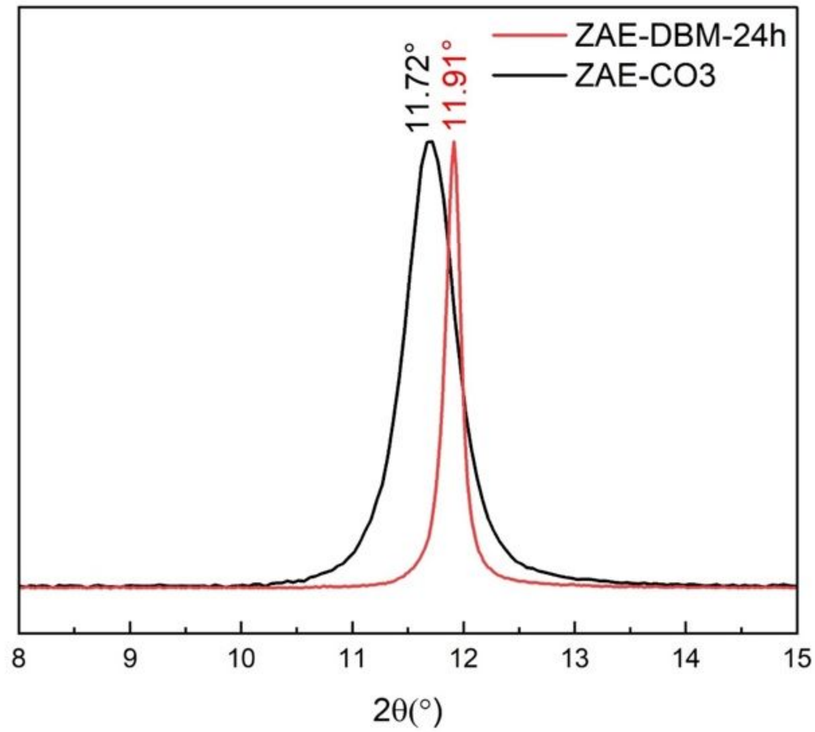

Figure S3. Normalized reflection (003) before (ZAE-CO3) and after memory effect (ZAE-DBM-24H). The basal spacing observed after rehydration (d003 ≈ 0.743 nm) is significantly different from that of the pristine LDH with intercalated carbonate (≈0.756 nm).

Table S1. Chemical analysis of LDHs

**Sample**	Zn (wt.%)	Al (wt.%)	Eu (wt.%)	Zn/(Al+Eu)	C (wt.%)	H (wt.%)	N (wt.%)
**ZAE5-CO3**	2.14	0.94	0.03	2.20	7.67	2.73	0.09
**ZAE5-460**	2.11	0.95	0.05	2.11	0.69	0.57	0.03
**ZAE-DBM-24h**	2.12	0.95	0.05	2.27	2.27	2.98	0.40
